# Editorial: Microbiome-gut-brain axis crosstalk and clinical outcomes

**DOI:** 10.3389/fnut.2024.1358659

**Published:** 2024-02-12

**Authors:** Xiaojie Zhang

**Affiliations:** Department of Psychiatry, Second Xiangya Hospital, Central South University, Changsha, China

**Keywords:** microbiome, gut, brain, MGB, clinical application, neuropsychiatric disease

Recently, intestinal microbiota is considered as a new and largest organ of the human body, with a total number of 10^13^-10^14^, ~10 times the number of human cells. The total number of bacterial genes encoded is 150 times the total number of human genes, known as the “second genome” of humans, and plays an important role in maintaining human health. It is a direct connection between external environment and human body. However, due to the existence of a blood-brain barrier between the central nervous system and the periphery, there is controversy over whether early gut microbiota affects brain function and diseases, resulting in research lagging behind other systemic diseases.

In recent years, an increasing number of studies have shown that gut microbiota disorders are closely related to various mental illnesses, including depression, bipolar disorder, schizophrenia, autism, attention deficit hyperactivity disorder, and substance related disorders. In 2012, Professor John F. Cryan and others from the University of Cork in Ireland emphasized the important role of Microbiome-Gut-Brain Axis (MGB). The gut microbiota can affect brain function and behavior through microbial derived metabolites, cytokines, immunity, vagus nerve pathways, and other pathways. The proposal of this concept not only enriches and develops the previous brain gut microbiota axis, but also provides a new entry point for revealing the pathogenesis of mental illness and exploring new diagnostic and treatment strategies.

This Research Topic “*Microbiome-gut-brain axis crosstalk and clinical outcomes”* takes advantage of the Frontiers publishing concept to advance communication among neuroscientists, microbiologist, geneticist, nutritionists, clinicians, psychiatrists, and psychologists with unique experience and skills, to fulfill the puzzle of host-microbiome interaction that contribute to health brain: introduces the correlation and possible mechanisms between gut microbiota disorders and the onset of various mental illnesses, as well as clinical and basic research on improving mental illnesses by regulating gut microbiota, in order to provide new directions for the etiology and diagnosis and treatment of mental illnesses.

In our topic, Murray et al. “*Demonstrating a link between diet, gut microbiota and brain: 14C radioactivity identified in the brain following gut microbial fermentation of 14C-radiolabeled tyrosine in a pig model*”, it proof that amino acids released from resistant protein following gut microbial fermentation, would be bioavailable to the brain (Figure 1). The original article “*Bifidobacterium animalis subsp. lactis and arginine mixture intake improves cognitive flexibility in mice*” by Ikuta et al., is the first report to show that probiotics consisting of *Bifidobacterium animalis* subsp. lactis and arginine (Bifal + Arg), improves cognitive flexibility in mice (Figure 2). A systematic review “*Potential neuroprotective effects of fermented foods and beverages in old age: a systematic review*” by Porras-García et al. also summarized the existing studies to establish whether the consumption of fermented foods and fermented beverages prevents or ameliorates neurodegenerative decline in old age. Overall, the evidence obtained in these articles not only enriches and develops the previous brain gut microbiota axis, but also provides a new technic for revealing the pathogenesis of mental illness and exploring new diagnostic and treatment strategies.

## Author contributions

XZ: Writing—original draft, Writing—review & editing.

